# Variation of Genes Encoding Tryptophan Catabolites Pathway Enzymes in Stroke

**DOI:** 10.3390/jcm8122133

**Published:** 2019-12-03

**Authors:** Paulina Wigner, Joanna Saluk-Bijak, Ewelina Synowiec, Elzbieta Miller, Tomasz Sliwinski, Natalia Cichon, Michal Bijak

**Affiliations:** 1 Laboratory of Medical Genetics, Faculty of Biology and Environmental Protection, University of Lodz, Pomorska 141/143, 90-236 Lodz, Poland; paulina.wigner@unilodz.eu (P.W.);; 2 Department of General Biochemistry, Faculty of Biology and Environmental Protection, University of Lodz, Pomorska 141/143, 90-236 Lodz, Poland; 3 Department of Neurological Rehabilitation, Medical University of Lodz, Poland, Milionowa 14, 93-113 Lodz, Poland; elzbieta.dorota.miller@umed.lodz.pl

**Keywords:** stroke, TRYCATs, tryptophan, polymorphism

## Abstract

The abnormal activation of the tryptophan catabolites pathway (TRYCATs) is observed in patients suffering from cerebrovascular disease, including stroke. A previous study confirmed that lower bioavailability of tryptophan for serotonin synthesis was characterized in the patients during the acute stroke phase. Interestingly, according to various studies, polymorphisms of the genes involved in the TRYCATs pathway may modulate the risk of stroke occurrence. Therefore, this study aimed to investigate the association between the occurrence of *TPH1, TPH2, KAT1, KAT2* and *IDO1* polymorphisms and the risk of stroke development.The following 10 polymorphisms of the genes encoding enzymes of the TRYCATs pathway were selected: c.804-7C > A (rs10488682), c.-1668T > A (rs623580), c.803+221C > A (rs1800532), c.-173A > T (rs1799913) – *TPH1*, c.-1449C > A (rs7963803), and c.-844G > T (rs4570625) – *TPH2*. c.*456G > A of *KAT1* (rs10988134), c.975-7T > C of *KAT2* (rs1480544), c.-1849C > A (rs3824259) and c. -1493G > C (rs10089084) of *IDO1*. The study was carried out on DNA isolated from the peripheral blood taken from 107 patients after a stroke and 107 healthy volunteers. All DNA samples were genotyped using TaqMan probes. The genotypes of eight studied polymorphisms modulated the risk of stroke. No significant difference in genotype and allele frequencies of the c.804-7C > A –*TPH1* (rs10488682) and c.*456G > A – *KAT1* (rs10988134) polymorphisms were found between patients and controls. Having performed haplotype and gen-gen analyses, it was possible to determine that patients after a stroke and controls differed in terms of the frequency of selected genotypes and haplotypes. Among the studied polymorphisms, eight SNPs were linked with stroke risk modulation. The results obtained confirmed our hypothesis regarding the involvement of the TRYCATs pathway in the pathogenesis of stroke.

## 1. Introduction

Stroke is considered and classified as a cardiovascular disease, next to coronary artery diseases, heart failure, hypertensive heart disease, valvular heart disease, and carditis [1]. The World Health Organization defines stroke as a “neurological deficit of cerebrovascular cause that persists beyond 24 h or is interrupted by death within 24 h” [2]. There were 6.5 million stroke deaths in the world in 2013, making stroke the second-leading cause of death behind ischemic heart disease. Approximately 795,000 strokes occur in the United States each year. Interestingly, someone has a stroke on average every 40 s in the United States, and someone dies of a stroke every 4 min [3]. There was an estimated number of 10.3 million new strokes in 2013, and 67% of them were ischemic strokes. Incidence for both ischemic and haemorrhagic strokes is higher among males than females [4]. Moreover, stroke is associated with high costs of treatment. It was estimated that the annual cost of treating CVD and strokes in the United States amounted to $316.1 billion from 2012 to 2013 [3]. Studies conducted in recent years demonstrated that the tryptophan catabolites pathway (TRYCATs) might be associated with the prevalence of cardiovascular diseases, including stroke [5,6,7]. Moreover, the activation of the TRYCATs pathway was increased after a stroke and may be linked with stroke severity and poor prognosis [7,8]. So far, research findings suggest that patients after an ischemic stroke have a lowered concentration of tryptophan and kynurenine acid. Tryptophan is an amino acid which is also a precursor to the neurotransmitter serotonin and hormone melatonin, while kynurenine acid is its neuroprotective metabolite and an antagonist of the N-methyl-D-aspartate (NMDA) receptor [9,10]. Moreover, the kynurenine/tryptophan (KYN/TRP) ratio, meaning kynurenine transaminase (KAT) activity, was lower, while the kynurenic acid/kynurenine (KYNA/KYN) ratio, meaning indoleamine-2,3-dioxygenase IDO activity, was higher in patients when compared to healthy volunteers [11].

IDO is a rate-limiting enzyme, which initiates tryptophan metabolism through O_2_-dependent oxidation, whereas KAT catalyses irreversible transamination of kynurenine acid in the brain and peripheral tissues [12,13,14]. Furthermore, an animal-based study confirmed that ischemia, which is observed during a stroke, increased the expression of TDO as compared to control animals [15]. Interestingly, the TRYCATs pathway is closely linked with inflammatory modulation. IDO may be activated by pro-inflammatory cytokines, whereas anti-inflammatory cytokines may act as inhibitors of the enzyme [16,17]. Thus, an inflammatory response to an acute ischemic stroke may cause the activation of IDO and, consequently, may lead to tryptophan depletion and increased generation of kynurenine [18]. Additionally, previous findings suggest that Interleukin 10 (IL-10) may reduce the capability of 5-TH synthesis in the brain, which is observed in patients after a stroke [19,20].

Moreover, Ormstad et al. [21] revealed that high levels of interleukin 1β are positively correlated with decreased bioavailability of TRP in the patients after an acute ischemic stroke. More details about the TRYCATs pathway were presented elsewhere [22]. The next study indicated that the activation of the kynurenine pathway plays a crucial role in the acute phase of a stroke as well as in fatigue and depression following a stroke. Patients with Fatigue Severity Scale (FSS) ≥4 at 12 months post-stroke, were characterized by a reduced tryptophan (TRP) index as compared to patients with FSS < 4 [21]. The index indicates the bioavailability of TRP in the plasma for serotonin (5-TH) synthesis in the brain [23]. Thus, the reduced TRP index confirmed lower bioavailability of TRP for 5-TH synthesis in patients during the acute stroke phase. Serotonin is important for wakefulness and inhibits rapid eye movement sleep [24]. Therefore, a low level of 5-TH synthesis may lead to abnormal tiredness and a need for long-lasting sleep [25].

In addition, an animal-based study showed that the level of kynurenine (a neurotoxic metabolite of tryptophan) during ischemia was increased in the brain [15]. Interestingly, kynurenine may activate an aryl hydrocarbon (Ah) receptor (ligand-activated transcription factor), and the activation is deleterious to cerebral ischemia. Accordingly, the study showed that the Ah receptor was overexpressed in the mouse brain after an experimental stroke, mainly in the neurons located at the peri-infarct and ischemic core. Similarly, increased expression of the Ah receptor was found in cultured rat cortical neurons after in vitro experimental ischemia [26]. Besides kynurenine, the level of another neurotoxic metabolite, i.e., quinolinic acid, was increased after transient ischemia in gerbils. This study was aimed to further elucidate the role of the TRYCATs pathway during a stroke in the Polish population. Therefore, single-nucleotide polymorphisms (SNPs) were investigated in the presented study. The studied SNPs were localized in the genes encoding the enzymes involved in the TRYCATs pathway, including tryptophan hydroxylase, kynurenine aminotransferase, and indoleamine 2,3-dioxygenase. Moreover, in this study, the further analysis included gen-gen interaction, haplotype and risk of stroke.

## 2. Materials and Methods

### 2.1. Subjects

We recruited 107 patients with stroke and 107 age- and sex-matched control subjects (without stroke) in our case-control study. The case group comprised patients after an ischemic stroke hospitalized at the Neurorehabilitation Department of the 3rd General Hospital in Lodz, Poland, in the years 2015–2017. The cerebral ischemic event in each patient had been documented using computed tomography (CT) of the brain. Neurological and CT findings were interpreted by two or more independent and experienced neurologists. All the patients had been diagnosed with an ischemic stroke; we did not include patients having other types of strokes.

Among the selected individuals, the people with a history of cranial trauma, cerebral haemorrhage, atrial fibrillation, other major sources of cardioembolism, coagulation disorders, tumours, chronic inflammatory diseases, and autoimmune diseases were excluded from the study. Having excluded all such cases, 107 subjects (50 males and 57 females) with a clinically overt stroke were selected and classified into the study group. Paresis of the left part of the body was observed more frequently (68 vs. 39).

A complete medical history was collected for all the individuals enrolled in the study; the medical history included smoking habits, alcohol consumption, presence of diabetes, and drug treatment. Hypertension was defined as either (based on a mean value of three independent measures) systolic blood pressure > 140 mm Hg, diastolic pressure > 90 mm Hg, or current treatment using antihypertensive drugs. Hypercholesterolemia was defined as either a need for statins or total plasma cholesterol level >200 mg/L. Diabetes was diagnosed when a given subject had a fasting glucose level >120 mg/L.

The healthy volunteer’s group included 107 healthy individuals with no history or prior diagnosis of any cardiovascular disease. The exclusion criteria were the same as those in the aforementioned study group. The controls were not related to the patients, and no history of stroke in their family members was confirmed.

All the patients were Caucasian and were recruited simultaneously from the same demographic area (central Poland). The full characteristics of the two groups are presented in Table 1.

All blood samples were collected in the morning (between 7 am and 9 am) in fasting status and stored using the same protocol. All procedures were done according to the Helsinki Declaration for Human Research and were approved by the Committee on the Ethics of Research in Human Experimentation at the University of Lodz with Resolution No. 28/2015. Informed consent was obtained from all participants and/or their legal guardians.

### 2.2. Selection of Single-Nucleotide Polymorphisms

All studied polymorphisms were chosen using the Database of Single Nucleotide Polymorphisms (dbSNP) of the National Center for Biotechnology Information (NCBI) available at http://www.ncbi.nlm.nih.gov/snp (Bethesda, MD, USA).

Our searches were conducted based on the following criteria:

1. a minor allele frequency of 0.05 or greater in the European population
2. location at the regulatory region of genes, i.e., the 5′ near a gene, the 5′ UTR, the 3′ UTR and intron. All studied polymorphisms are presented in Table 2.

### 2.3. DNA Isolation

Genomic DNA was extracted from venous blood according to the SaMag™ Blood DNA Extraction Kit protocol and using the commercially available SaMag™ System (Sacace Biotechnologies S.r.l., Vicenza, Como, Italy). Then, the concentration and purity of DNA samples were determined by a spectrophotometer, where a 260/280 nm optical density (OD) ratio in range 1.8–2.0 was considered high quality and stored at −20 °C until use. the Genotyping.

**Figure 1:**
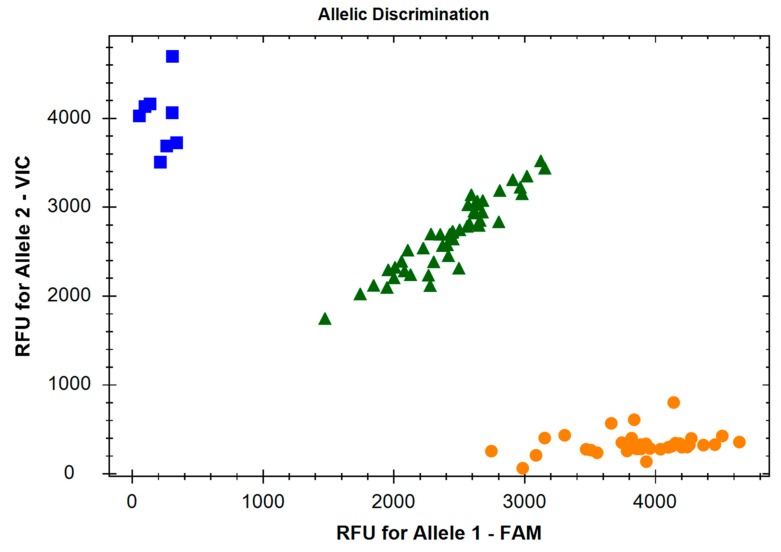
Allelic discrimination X-Y scatter-plot of the c.804-7C>A SNP (rs1799913) of the *TPH1* gene. The TaqMan^®^ SNP Genotyping Assay (ID: C___2645661_10) was used for genotyping of this SNP. The *χ*-axis represents the relative fluorescent emission for the A allele-specific probe labelled with 6-carboxyfluorescein (FAM), and the *Ɣ*-axis represents the emission for the C allele-specific probe labelled with 2’-chloro-7’-phenyl-1,4-dichloro-6-carboxyfluorescein (VIC). Circles: homozygous A/A; squares: homozygous C/C; triangles: heterozygous C/A.

During our study, we investigated 10 SNPs of the genes encoding enzymes of the TRYCATs pathway. TaqMan^®^ SNP Genotyping Assays (Thermo Fisher Scientific, Waltham, Massachusetts, USA) and TaqMan™ Universal Master Mix II, no UNG (Thermo Fisher Scientific, Waltham, Massachusetts, USA) were used to perform the genotyping of the selected SNPs. The Taq-Man Assay IDs are presented in Table 3. The thermal cycling conditions were as follows: initial denaturing at 95 °C for 10 min, 40 cycles of 92 °C for 15 sec and 60 °C for 60 sec. All reactions were carried out in a thermal cycler CFX96™ Real-Time Polymerase Chain Reaction (PCR) Detection System (BIO-RAD). The genotypes were determined automatically based on dye-component fluorescent emission data depicted in the X-Y scatter-plot of the CFX Manager TM Software (version 3.1- BIO-RAD, Hercules, California, USA). The Figure 1 shows a representative allelic discrimination X-Y scatter-plot of the c.804-7C>A SNP (rs1799913) of the TPH1 gene.

### 2.4. Statistical Analysis

A statistical analysis of the results was performed in Statistica, version 12 for Windows (Statsoft, Tulsa, OK, USA), and SigmaPlot, version 11.0 for Windows (Systat Software Inc., San Jose, CA, USA). Calculations of the association between case/control and each studied polymorphism were performed in accordance with the unconditional multiple logistic regression model. The odds ratios (ORs) with 95% confidence interval (95% CI) served to assess the strength of association. Haplotypic frequencies were estimated using MedCalc software, version 15.0 for Windows (Ostend, Belgium). The values of *p* < 0.05 were considered statistically significant.

## 3. Results

### 3.1. Single Nucleotide Polymorphisms of the Genes Encoding TRYCATs Enzymes (TPH1, TPH2, KAT1, KAT2 and IDO1) as the Risk of Stroke Occurrence

Among the studied SNPs (Table 4), the heterozygote and the C allele of c.804-7C > A *– TPH1* (rs1799913) were associated with a reduced risk of stroke occurrence, and the homozygote A/A and the A allele of the same SNP decreased this risk. A statistical analysis of disturbances in the genotypes and alleles of the c.803 + 221C > A *– TPH1* (rs1800532) polymorphism confirmed that the C/C and A/A homozygotes were linked with an increased frequency of stroke occurrence by 20 times, while the heterozygote of the same SNP showed a protective character and reduced the risk. The results demonstrated that the T/T genotype and T allele of c.-1668T > A *– TPH1* (rs623580) were negatively correlated with stroke, whereas the A/A, T/A and A allele of the same polymorphism were positively correlated with the disease. Interestingly, the analysis of distribution of the genotypes and alleles of the c.-844G > T *– TPH2* (rs4570625) polymorphism showed that the G/G homozygote and the G allele increased the risk of stroke by more than 30 times (*p* < 0.001), while the G/T genotype of the same SNP decreased the risk by nearly 27 times (*p* < 0.001). In case of c.-1449C > A *– TPH2* (rs7963803), the C/C genotype and the C allele were positively correlated with stroke occurrence, whereas the heterozygote and the A allele protected against the onset of stroke. Additionally, the c.975-7T > C – *KAT2* (rs1480544) polymorphism modulated the risk of stroke development. The C/C genotype and the C allele of the SNP increased the risk of the disease, while the T/C and the T allele reduced the risk. Moreover, in the case of the c.-1493G > C – *IDO1* (rs10089084) polymorphism, the G/G genotype and the G allele were linked with an increased frequency of stroke occurrence as compared to the control subjects, whereas the C/C homozygote and the C allele reduced this risk. No correlation was found between genotypes/alleles of the c.-173A > T – *TPH1* (rs10488682), c.*456G > A – *KAT1* (rs10988134), c.-1849C > A – *IDO1* (rs3824259) polymorphisms and stroke development.

### 3.2. Haplotypes and DD Prevalence

Our team also investigated the correlation between the occurrence of stroke and haplotypes (Table S1) of the c.804-7C > A (rs1799913) and c.803+221C > A (rs1800532), c.804-7C > A (rs1799913) and c.-173A > T (rs10488682), c.804-7C > A (rs1799913) and c.-1668T > A (rs623580), c.-173A > T (rs10488682) and c.803+221C > A (rs1800532), c.-1668T > A (rs623580) and c.803+221C > A (rs1800532), c.-173A > T (rs10488682) and c.-1668T > A (rs623580) polymorphisms of the gene encoding *TPH1*, c.-1449C > A (rs7963803) and c.-844G > T (rs4570625) SNPs of the *TPH2* gene, c.-1849C > A (rs3824259) and c.-1493G > C (rs10089084) SNP of the *IDO1* gene. In case of c.804-7C > A (rs1799913) and c.803+221C > A (rs1800532), haplotype AC decreased the risk of stroke by more than seventy-six times (*p* < 0.0001), while haplotype AA of the same SNPs combination only doubled this risk. Moreover, the CT haplotype of c.804-7C > A (rs1799913) and c.-173A > T (rs10488682) SNPs reduced the risk of stroke occurrence, whereas AT of the same combination of polymorphisms increased this risk. The obtained results showed that the TT and AT haplotypes of the c.804-7C > A (rs1799913) and c.-1668T > A (rs623580) polymorphisms were negatively correlated with stroke development, whereas the CA haplotype of the same combined SNPs increased the risk of the disease. Additionally, the AA haplotype of the c.-173A > T (rs10488682) and c.803+221C > A (rs1800532) combined SNPs reduced the frequency of stroke in the Polish population as compared to healthy volunteers. In the case of the c.-1668T > A (rs623580) and c.803+221C > A (rs1800532) polymorphism, the TC haplotype was linked with the reduced risk of stroke development, while the AC haplotype of the same combination increased the frequency of stroke occurrence. Moreover, the AA and TA haplotypes of c.-173A > T (rs10488682) and c.-1668T > A (rs623580) were positively correlated with stroke, whereas the AT and TT haplotypes negatively correlated with the disease. In case of combined polymorphisms encoding *TPH2* – i.e., c.-1449C > A (rs7963803) and c.-844G > T (rs4570625) – the CG increased the risk of stroke, while the CT, AG and AT haplotypes reduced this risk. Besides, the CG haplotype of c.-1849C > A (rs3824259) and c.-1493G > C (rs10089084) was linked with an increase in the risk of stroke occurrence, whereas the CC haplotype of the same SNPs combination reduced the risk.

### 3.3. Gene-Gene Interactions and the Risk of Stroke

We also investigated the correlation between stroke occurrence and combined genotypes of all the studied polymorphisms (Table S2). We found that the C/C-G/G combined genotype of the c.804-7C>A *– TPH1* (rs1799913) and c.-844G > T *– TPH2* (rs4570625) polymorphisms were associated with an increased risk of stroke. Interestingly, the presence of the C/A-G/T genotype of the same polymorphism combination reduced the risk of stroke development 100 times (*p* < 0.001), whereas the A/A-G/G combined polymorphisms elevated this risk more than 50 times (*p* < 0.001). There was a link between an increased risk of stroke and the frequency of the C/C-C/C, A/A-C/C genotypes of c.804-7C > A *– TPH1* (rs1799913) and c.-1449C > A *– TPH2* (rs7963803), while the C/A-C/C and A/A-C/A genotypes reduced this risk. In the case of c.803 + 221C > A *– TPH1* (rs1800532) and c.-844G > T *– TPH2* (rs4570625), the C/C-G/G and C/A-G/G were linked with the risk of stroke elevated by more than 10 times (*p* < 0.001). On the other hand, the C/A-G/T genotypes of the same combined genotypes reduced this risk. Moreover, we observed that the C/C-C/C combined genotype of the c.803 + 221C > A *– TPH1* (rs1800532) and c.-1449C > A *– TPH2* (rs7963803) polymorphisms were associated with an increased risk of stroke, whereas the C/A-C/A genotypes reduced the risk. Interestingly, the A/A-C/C genotypes of the same polymorphism combination increased the occurrence of stroke by more than 18 times (*p* = 0.005). The T/T-G/G and A/T-G/G genotypes of c.-173A > T *– TPH1* (rs10488682) and c.-844G > T *– TPH2* (rs4570625) SNPs caused an above 30-fold (*p* < 0.001) and a nearly 70-fold (*p* < 0.001) increase of the risk in the Polish population, respectively. However, the T/T-G/T and A/T-G/T genotypes of the same SNP combination were associated with a diminished risk of stroke occurrence. Moreover, we found that the T/T-C/C genotype of c.-173A > T *– TPH1* (rs10488682) and c.-1449C > A *– TPH2* (rs7963803) was linked with an elevated risk of stroke development, whereas the A/T-C/A genotype caused a decrease of this risk by nearly fifteen times. In the case of c.-1668T > A *– TPH1* (rs623580) and c.-844G > T *– TPH2* (rs4570625), combined genotypes T/T-G/G and T/A-G/G were associated with a more than 10-fold (*p* < 0.001) and eleven-fold (*p* < 0.001) increase of the DD risk, respectively. On the other hand, the T/T-G/T and T/A-G/T genotypes of the same combinations diminished the risk of the disease more than 20-fold (*p* < 0.001) and more than twofold (*p* = 0.004), respectively Additionally, the presence of the T/T-C/C and T/A-C/A combined genotypes of the c.-1668T > A *– TPH1* (rs623580) and c.-1449C > A *– TPH2* (rs7963803) polymorphisms reduced the risk of stroke development, whereas the T/A-C/C and A/A-C/C genotypes were linked with an increased risk of the disease. Furthermore, we revealed that the G/G-C/A genotype of c.*456G > A (rs10988134) – *KAT1* and c. -1849C > A – *IDO1* (rs3824259) increased the risk of stroke. In addition, we observed that the C/C-C/A genotype of c.975-7T > C– *KAT2* (rs1480544) and– c.-1849C > A *IDO1* (rs3824259) SNPs was associated with an increased risk of stroke. However, the T/C-C/A and T/C-A/A genotypes of the same combination caused an increase of stroke occurrence by more than 30 times (*p* < 0.001) and six times (*p* = 0.005), respectively. In the case of the c.-1493G > C – *IDO1* (rs10089084) and c.*456G > A– *KAT1* (rs10988134) combined genotypes, we found that G/G-G/G and G/G-G/A were associated with a more than fourfold increase of the stroke risk, while the C/C-G/G combined genotype of the same polymorphisms was associated with an increase of this risk by about 10 times (*p* < 0.001). The link between the reduced risk of stroke and the frequency of the G/G-T/C and G/A-T/C genotypes of the c.*456G>A– *KAT1* (rs10988134) and c.975-7T>C– *KAT2* (rs1480544) polymorphisms were confirmed in our study. On the other hand, we found that the G/G-C/C and G/A-C/C genotypes of the same SNP combination caused an increase in the frequency of stroke occurrence. Moreover, we found that the G/G-T/T, G/G-C/C and G/C-C/C genotypes of c.-1493G>C – *IDO1* (rs10089084) and c.975-7T > C– *KAT2* (rs1480544) increased the risk of stroke about four times (*p* = 0.009), 18 times (*p* < 0.001) and three times (*p* < 0.001), respectively. On the other hand, the G/C-T/C genotype of the same polymorphism caused a drop in the risk just about fourteen times (*p* < 0.001). In the case of the c.-1849C > A – *IDO1* (rs3824259) and c.804-7C > A – *TPH1* (rs1799913) polymorphisms, the C/C-C/A and C/A-C/A combined polymorphisms were linked with a reduction of stroke risk about 20 times (*p* = 0.004) and 10 times (*p* < 0.001), respectively. The C/C-G/T, C/A-G/T and A/A-G/T combined genotypes of c.-1849C>A – *IDO1* (rs3824259) and c.-844G > T – *TPH2* (rs4570625) caused a reduction of the stroke risk in the Polish population. However, the C/C-G/G, C/A-G/G and A/A-G/G genotypes of the same combined SNPs led to an increase of this risk about five times (*p* = 0.003), 66 times (*p* < 0.001) and 10 times (*p* < 0.001), respectively. In the case of the next combined polymorphism, i.e., c.-1849C > A – *IDO1* (rs3824259) and c.803+221C>A – *TPH1* (rs1800532), we confirmed that the C/C-C/A and A/A-C/A genotypes decreased the risk of stroke occurrence, while the C/A-C/C and A/A-A/A genotypes increased this risk in the studied population. Moreover, the frequency of the C/C-C/A genotype of c.-1849C > A – *IDO1* (rs3824259) and c.-1449C>A - *TPH2* (rs7963803) was decreased, while the C/A-C/C genotype was increased in the patients after a stroke as compared to the healthy volunteers. Additionally, we found that the only C/A-T/T genotype of the c.-1849C > A – *IDO1* (rs3824259) and c.-173A>T – *TPH1* (rs10488682) combined polymorphisms elevated the risk of stroke development. The C/C-T/T and C/A-T/T genotypes of c.-1849C>A – *IDO1* (rs3824259) and c.-1668T > A – *TPH1* (rs623580) were associated with a reduced risk of stroke, whereas the C/A-T/A and C/A-A/A genotypes caused an increase of the risk in the Polish population. Interestingly, the G/G-C/C, G/G-A/A as well as G/C-A/A of the c.-1493G > C – *IDO1* (rs10089084) and c.804-7C > A – *TPH1* (rs1799913) combined polymorphisms caused an increased frequency of stroke occurrence in the Polish population more than 20 times (*p* = 0.003), five times (*p* < 0.001), and nearly four times (*p* < 0.001), respectively. However, the G/C-C/A genotype of the same polymorphism combination was associated with increased stroke risk in this population by about eight times (*p* < 0.001). On the one hand, we observed that the G/C-G/G combined genotype of the c.-1493G > C – *IDO1* (rs10089084) and c.-844G > T – *TPH2* (rs4570625) polymorphisms were associated with an increased risk of stroke. On the other hand, we found that the G/C-G/T and C/C-G/T genotypes of the same combined SNPs caused a reduction of this risk about six times (*p* < 0.001) and 17 times (*p* < 0.001), respectively. Interestingly, in the case of c.-1493G>C – *IDO1* (rs10089084) and c.803+221C > A – *TPH1* (rs1800532), we observed that the presence of the G/G-C/C combined genotype elevated the risk of stroke development more than 22 times (*p* = 0.003), whereas the C/C-C/A genotype was associated with a nearly 18-fold increase of the risk in the Polish population (*p* < 0.001). Moreover, an elevated risk of stroke for the G/G-C/G genotypes and a reduced risk for the G/C-C/A and C/C-C/C genotypes of the same polymorphisms were confirmed in our study. In our study, we confirmed the link between a reduced risk of stroke and the frequency of the C/C-C/C genotype of the c.-1493G>C – *IDO1* (rs10089084) and c.-1449C > A – *TPH2* (rs7963803) polymorphisms. However, the G/G-C/C and G/C-C/C genotypes of the same polymorphism combination increased this risk in the studied polymorphism. The G/G-A/T and G/G-T/T genotypes of c.-1493G>C – *IDO1* (rs10089084) and c.-173A > T – *TPH1* (rs10488682) caused an increase of the risk of stroke, whereas the C/C-A/T and C/C-T/T genotypes of the same combined polymorphisms brought about a reduction of this risk. We found that the G/C-T/T, C/C-T/T and C/C-T/A genotypes of c.-1493G > C – *IDO1* (rs10089084) and c.-1668T>A – *TPH1* (rs623580) reduced the risk of stroke, whereas the G/G-T/A genotype increased this risk. Moreover, the frequency of the G/G-C/A and G/A-C/A genotypes of the c.*456G > A (rs10988134) – *KAT1* and c.804-7C > A – *TPH1* (rs1799913) combined polymorphisms was decreased in the patients after a stroke as compared to the controls by about 12 times (*p* < 0.001) and 25 times (*p* = 0.002), respectively. However, in the case of the same polymorphisms, the patients with stroke were characterized by an increased frequency of occurrence of the G/G-A/A, G/A-C/C and G/A-A/A genotypes. Moreover, the G/G-G/G combined genotypes of c.*456G > A (rs10988134) – *KAT1* and c.-844G > T – *TPH2* (rs4570625) caused a more than elevenfold increase of the risk in the Polish population (*p* < 0.001), whereas the G/G-G/T and G/A-G/T genotypes caused a reduction of stroke occurrence. We also confirmed that the G/G-C/A and G/A-C/A combined genotypes of c.*456G > A (rs10988134) – *KAT1* and c.803+221C > A – *TPH1* (rs1800532) were related with a decreased risk of stroke, but the G/G-A/A and G/A-C/C combined genotypes of the same polymorphism increased this risk. The G/G-C/C, G/A-C/C combined genotypes of c.*456G>A (rs10988134) – *KAT1* and c.-1449C > A – *TPH2* (rs7963803) were linked with elevated occurrence of stroke, while the G/G-C/A genotype of the same polymorphism combination decreased this risk more than thirty times (*p* < 0.001). Additionally, the presence of the G/G-T/T and G/A-T/T combined genotypes of the c.*456G > A (rs10988134) – *KAT1* and c.-1668T > A – *TPH1* (rs623580) polymorphisms reduced the risk of stroke development, while the G/G-T/A genotype increased this risk in the studied population. The T/T-A/A, C/C-C/C and C/C-A/A genotypes of c.975-7T > C– *KAT2* (rs1480544) and c.804-7C > A - *TPH1* (rs1799913) caused an increase of the risk of stroke. Interestingly, the presence of the T/T-G/G and C/C-G/G combined genotypes of the c.975-7T > C– *KAT2* (rs1480544) and c.-844G>T – *TPH2* (rs4570625) polymorphisms elevated the risk of stroke development more than 30 times (*p* = 0.001). On the other hand, the T/T-G/T genotype of the same combined SNPs caused a decrease of this risk. Moreover, the T/C-C/A combined genotypes c.975-7T > C– *KAT2* (rs1480544) and c.803+221C > A – *TPH1* (rs1800532) caused a more than 20-fold reduction of the stroke risk in the Polish population (*p* = 0.001), whereas the C/C-C/C genotype increased this risk about ten times (*p* = 0.001). The T/T-C/C and C/C-C/C genotypes of c.975-7T > C – *KAT2* (rs1480544) and c.-1449C > A – *TPH2* (rs7963803) caused an increase of the risk of DD by just about three times (*p* = 0.003) and 11 times (*p* < 0.001), respectively. However, the T/C-C/C and C/C/C/A genotypes of the same combined polymorphisms brought about a reduction of this risk by 16 times (*p* < 0.001) and 13 times (*p* = 0.014), respectively. The T/T-T/T, C/C-A/T, C/C-T/T combined genotypes of c.975-7T > C– *KAT2* (rs1480544) and c.-173A > T – *TPH1* (rs10488682) were linked with an increased risk of stroke occurrence, while the T/C-T/T genotype of the same polymorphism combination decreased this risk. Moreover, we found that the T/T-T/A, C/C-T/A, C/C-A/A genotypes of c.975-7T > C– *KAT2* (rs1480544) and c.-1668T > A – *TPH1* (rs623580) increased the risk of stroke about threefold (*p* = 0.016), fourfold (*p* < 0.001) and 27-fold (*p* < 0.001), respectively. On the other hand, the T/C-T/T and T/C-T/A genotypes of the same combined SNPs caused a drop in stroke occurrence by more than 37 times and 15 times, respectively. No statistical correlation was found between combined genotypes of the c.*456G > A (rs10988134) – *KAT1* and c.-173A > T – *TPH1* (rs10488682) polymorphisms and the development of stroke.

## 4. Discussion

A growing body of evidence suggests that abnormal tryptophan metabolism may play a crucial role in stroke development. Our results presented in this paper confirmed this hypothesis. This is the first study to show that all chosen polymorphisms of the genes encoding enzymes of the TRYCATs pathway (i.e., tryptophan hydroxylases, kynurenine aminotransferases, indoleamine 2,3-dioxygenases) may modulate the risk of stroke occurrence.

Tryptophan hydroxylase, which exists in two isoforms: TPH1 and TPH2, is one of the key enzymes of tryptophan metabolism. Human *TPH1* is located at chromosome 11p15.3 – 14, and comprises 11 exons, while the human TPH2 gene is located at chromosome 12q15 and comprises 11 exons. Both isoforms are highly homologous and exhibit 71% of amino acid identity [27]. TPH converts L-tryptophan into L-5-hydroxytryptophan (serotonin precursor) [28]. Thus, abnormal amounts or activity of this enzyme may cause deficiencies of neuroprotective compounds such as kynurenic acid, and consequently, lead to the development of several diseases, including stroke. In our study, we genotyped four polymorphisms of *TPH1* and two SNPs of *TPH2.* One of them – c.804 – 7C > A *– TPH1* (rs1799913) – is localised at intron 7 and the polypyrimidine stretch immediately upstream of the 3′acceptor splice site. This substitution of pyrimidine for purine in the polypyrimidine consensus sequence may decrease the fidelity of splicing. Indeed, sequencing of TPH1 cDNA revealed no evidence of abnormal splicing [29]. So far, studies showed that the SNP was linked with increased 5-hydroxy indole acetic acid concentrations in cerebrospinal fluid [30]. Thus far, this SNP was only investigated in affective disorders. Previous studies showed that the C/C genotype of this SNP was associated with the development of depression [22,31]. By contrast, we found that the C/A genotype may elevate, while the A/A genotype of the same genotype may increase the risk of stroke occurrence. This disturbance may be a result of differences in the activation of the TRYCATs pathway during the development of depression and stroke. Moreover, the number of case and control groups in both studies differed. The next potentially functional TPH1 polymorphism in our study is the c.803 + 221C > A polymorphism of TPH1 (rs18005832). This SNP is localised at intron 7 in the potential GATA transcription factor-binding site, which allows the initiation of transcription. A previous study demonstrated that the polymorphism might be involved in the pathophysiology of affective disorders [22]. The A/A genotype may increase the risk of depression development in the Polish population. Similarly, in the case of the *TPH1* polymorphism, we confirmed that the A/A genotype increased the risk of stroke. Moreover, the frequency of A allele was high in suicide attempters as compared to healthy volunteers in a Turkish population [32]. However, the same polymorphism was not associated with the occurrence of schizophrenia in Japanese populations [33]. Interestingly, this polymorphism was also linked with an elevated alcohol dependence risk in a Caucasian population, whereas the association was not of significance in Asian populations [34]. Another *TPH1* polymorphism studied during this experiment was c.-173A > T (rs10488682). The SNP is localized in the promoter region of *TPH1* and may reduce promoter activity, changing the transcription level of *TPH1* [35]. So far, we have been the first to investigate that c.-173A > T SNP of *TPH1* may contribute to the development of stroke. We found that the polymorphism was not associated with the occurrence of stroke. Likewise, the case-control study revealed no statistically significant association between *TPH1* SNPs and progression of idiopathic scoliosis in an Eastern European population [36]. On the other hand, the A allele of the polymorphism was associated with a tendency to be resistant to brace treatment of adolescent idiopathic scoliosis in an Asian population [37]. Moreover, Wigner et al. [22] showed that the T/T genotype caused an increase in depression risk.

The next studied polymorphism was c.-1668T > A SNP (rs623580). The SNP is localised in the exon 1c/intron 1 region and is within the 5′UTR region [38]. The T/T genotype of this polymorphism was associated with a decreased risk of depression, while the A/A genotype increased this risk [22]. Similarly, in our study, we confirmed that T/T increased the risk of stroke occurrence, and the A/A genotype elevated this risk as well. Moreover, Kwak et al. [39] found that this polymorphism was linked with the body mass index, a measure of obesity frequently related to MDD.

Besides the SNP localised in *TPH1*, we also studied *TPH2* polymorphisms, and c.-844G>T (rs4576025) was one of them. This polymorphism is associated with a change of DNA-protein interactions, ultimately affecting transcription of the *TPH2* gene. Indeed, the presence of the T allele may lead to a reduction of *TPH2* promoter activity and may cause inhibition of serotonin synthesis [40,41,42,43]. Additionally, the SNP demonstrates a potential impact on the modulation of amygdala’s response to emotional stimuli [44,45,46]. Interestingly, the amygdala’s response may also be increased by the reduction of serotonin signals [47,48]. Thus, the SNP may modulate emotion signalling through impairment of the serotonin function. Previous studies showed that the G/G genotype of the polymorphism was associated with an increased risk of depression and suicidal attempts in depressed patients. Moreover, the heterozygote of this polymorphism was negatively correlated with depression occurrence [22]. Similarly, we found that the G/G homozygote was positively correlated with stroke occurrence, whereas the G/T genotype caused a reduction of this risk.

Interestingly, stroke survivors were characterized by the occurrence of affective disorders both in the acute period and follow-ups, which may adversely impact rehabilitation and prognosis [49]. Thus, previous studies suggest that the TRYCATs pathway is involved in the pathophysiology of post-stroke anxiety [50,51]. Moreover, the G allele of the *TPH2* polymorphism was associated with the occurrence of post-stroke anxiety [52]. Likewise, our study confirmed that the G allele of this SNP caused an increase in the stroke risk. Furthermore, the SNP of the *TPH2* gene was associated with the development of paranoid schizophrenia, multiple sclerosis, and panic disorders [37,46,53]. The second studied polymorphism of *TPH2* was c.-1449C > A (rs7963803). Moreover, the C/C homozygote and C allele of this SNP decreased the risk of development of depressive disorders [22]. On the other hand, our study showed that this homozygote increased the risk of stroke occurrence, whereas the C/A heterozygote reduced this risk. Such disturbances may be a result of different bases for the development mechanism of both diseases or varied sizes of the studied groups.

Kynurenine aminotransferase is the next key enzyme of the TRYCATs pathway, described in this paper. KAT catalyses irreversible transamination of kynurenine acid in the brain and peripheral tissues [13,14]. Four isoforms of this enzyme have been described so far (KATI/glutamine transaminase K (GTK)/cysteine conjugate beta-lyase (CCBL) 1, KATII/aminoadipate aminotransferase (AADAT), KAT III/CCBL2, and KAT IV/glutamicoxaloacetic transaminase (GOT) 2/mitochondrial aspartate aminotransferase (ASAT) [54]. However, we studied only two isoforms, i.e., KAT1 and KAT2. The gene encoding KAT1 is localized at chromosome 9q34.11; this enzyme limits the generation of kynurenic acid. We were the first to investigate the link between the c.*456G > A (rs10988134) polymorphism of *KAT1* and stroke occurrence. This SNP causes a transition in the 3′UTR region, which may affect *KAT1* transcript stability [55]. To date, several transcript variants of *KAT1,* which are known to influence substrate specificity, have been described. We found no link between polymorphism occurrence and the risk of stroke. Interestingly, apart from our team, no one has been concerned with this polymorphism so far. *KAT2* is another isoform of KAT presented in this paper. The gene of this enzyme is localized on chromosome 4q33, while the studied c.975-71T > C (rs1480544) polymorphism is found in putative exonic splicing silencers (ESSs). The SNP may cause quantitative changes in the production of canonical mRNAs and peptide generation, and, consequently, may contribute significantly to inter-individual phenotypic variability [56]. Moreover, the C allele may cause an increase in mRNA expression and KATII protein production [57]. Thus, in our study, we found that the C/C genotype of this polymorphism caused an increase of the stroke risk, while the C/T genotype decreased this risk. Additionally, a previous study, which had been conducted on a group of patients with human immunodeficiency virus (HIV), revealed that the C allele of this polymorphism could protect against psychopathological distress in controls, but not in HIV individuals [58].

Indoleamine 2,3-dioxygenase, which catalyses the first and rate-limiting step in the TRYCATs pathway leading to the initiation of N-formylkynurenine, is yet another enzyme involved in the transformation of tryptophan. The gene encoding this enzyme is localized at chromosome 8p11 [12,59,60]. Furthermore, an animal-based study confirmed that ischemia, which is observed during a stroke, increased the expression of tryptophan 2,3-dioxygenase (TDO) as compared to control animals. TDO may also catalyse the first and rate-limiting step of tryptophan degradation along the kynurenine pathway [15]. Interestingly, the TRYCATs pathway is closely linked with inflammatory modulation. IDO may be activated by pro-inflammatory cytokines, whereas anti-inflammatory cytokines may act as inhibitors of the enzyme [16,17]. Thus, an inflammatory response to an acute ischemic stroke may cause the activation of IDO, and, consequently, may lead to tryptophan depletion and increased generation of kynurenine [18]. Additionally, previous findings suggest that Interleukin 10 (IL-10) may reduce the capability of 5-TH synthesis in the brain, which is observed in the patients after a stroke [19,20]. Moreover, Ormstad et al. [21] revealed that high levels of interleukin 1β are positively correlated with decreased bioavailability of TRP in the patients after an acute ischemic stroke. More details about the TRYCATs pathway were presented somewhere else [22]. In presented paper the both studied polymorphisms of *IDO1*, i.e., c.-1849C > A (rs3824259) and c.-1493G > C (rs10089084), are localized in 5′ region of the gene. A previous study showed no association between the two polymorphisms and IFN-α-related depression [61]. Similarly, our team found no correlation between the frequency of the c.-1849C > A (rs3824259) polymorphism and the risk of stroke occurrence. On the other hand, in case of the second studied polymorphism, c. -1493G > C (rs10089084), we demonstrated that the G/G homozygote was associated with an increased risk of stroke development, whereas the C/C genotype decreased this risk.

The findings of this work cast a new light on the pathogenesis of stroke; however, the present study had some potential limitations. The size of the studied group was relatively small and consequently of low power, which could lead to both false negative as well as false-positive findings. Thus, received results should be interpreted with caution and considered preliminary. Moreover, the studied population included patients and controls from Poland only, which reduces the possibility of confounding from ethnicity, so it does not permit any extrapolation of the results to other ethnic groups. Therefore, some additional larger case-control studies on different population groups and functional experiments are necessary before the final resolution about/findings as to the role of the TRYCATs pathway in the development of this disease. Moreover, the next limitations included no matching of the control group and patients in terms of vascular risk factors. In the future, the analysis should be extended to both the control group/patients with and without this risk. To conclude, the presented study showed a significant association between the tryptophan catabolism pathway and the occurrence of stroke, but the results obtained should be interpreted with great caution.

## 5. Conclusions

In the presented paper, we confirmed that the selected SNPs of the genes involved in the tryptophan catabolites pathway might influence the risk of depression occurrence. Among the studied SNPs, we found no link between the risk of stroke occurrence and only two polymorphisms, c.-173A > T – *TPH1* (rs10488682) and c.*456G > A – *KAT1* (rs10988134). This study confirms the hypothesis that the TRYCATs pathway may be involved in the pathogenesis of stroke development. Thus, these polymorphisms may be considered independent markers of stroke. SNPs as biomarkers will allow the use of preventive measures in people at increased risk of stroke occurrence. Moreover, these markers will allow quick and new therapeutic strategies. In the future, further molecular studies of the underlying causes of stroke, including analyzing the level of expression of the studied genes, are necessary.

## Figures and Tables

**Table 1:** Demographic and clinical data in patients with history of ischemic stroke and controls.

	Patients (*n* = 107)	Control (*n* = 107)	*p*
Age	50.1 ± 11.8	47.3 ± 8.7	0.049 *
Gender (M/F)	50/57	53/54	0.7844
Hypertension	83	54	0.0001
Hypercholesterolemia	77	52	0.0008
Diabetes	36	24	0.0941
Smoking (current)	37	35	0.8850
Smoking (former)	20	20	0.8608
Daily alcohol consumption	27	23	0.6279

*p* - values for a two-sided χ^2^ - test; except for * for *t*-test.

**Table 2:** Characteristics of studied single-nucleotide polymorphisms (SNPs).

Gene	rs Number	Polymorphism	Localization
***TPH1***	rs1799913	c.804-7C>A	intron
	rs623580	c.-1668T>A	near gene 5′
	rs1800532	c.803 + 221C>A	intron
	rs10488682	c.-173A>T	near gene 5′
***TPH2***	rs7963803	c.-1449C>A	near gene 5′
	rs4570625	c.-844G>T	
***KAT 1***	rs10988134	c.*456G>A	UTR-3′
***KAT 2***	rs1480544	c.975-7T>C	intron
***IDO 1***	rs3824259	c.-1849C>A	near gene 5′
	rs10089084	c. -1493G>C	

**Table 3:** Details of TaqMan^®^ SNP genotyping assays used in this study.

Polymorphism	Assay ID	Location
rs1799913	C___2645661_10	Chr.11: 18,025,708 on GRCh38
rs623580	C___2645676_10	Chr.11: 18,042,430 on GRCh38
rs1800532	C___8940793_10	Chr.11: 18,026,269 on GRCh38
rs10488682	C___2645675_10	Chr.11: 18,040,935 on GRCh38
rs7963803	C__27855793_30	Chr.12: 71,937,538 on GRCh38
rs4570625	C____226207_10	Chr.12: 71,938,143 on GRCh38
rs10988134	C__11840549_10	Chr.9: 128,833,128 on GRCh38
rs1480544	C___8242555_10	Chr.4: 170,066,485 on GRCh38
rs3824259	C__27491530_10	Chr.8: 39,912,074 on GRCh38
rs10089084	C__30475151_10	Chr.8: 39,912,430 on GRCh38

**Table 4:** Distribution of genotypes and alleles of c.804-7C > A – *TPH1* (rs1799913), c.803+221C > A – *TPH1* (rs1800532), c.-173A > T – *TPH1* (rs10488682), c.-1668T > A – *TPH1* (rs623580), c.-844G > T – *TPH2* (rs4570625), c.-1449C > A – *TPH2* (rs7963803) c.*456G > A of *KAT1* (rs10988134), c.975-7T > C of AADAT (rs1480544), c.-1849C > A (rs3824259) and c.-1493G > C (rs10089084) of *IDO1* and incidence of stroke.

Genotype/Allele	Control (*n* = 107)		Stroke (*n* = 107)		Crude OR (95% CI)	*p*
	Number	Frequency	Number	Frequency		
c.804-7C > A – *TPH1* (rs1799913)						
C/C	23	0.215	35	0.327	1.775 (0.962–3.277)	0.066
C/A	57	0.533	5	0.047	0.043 (0.016–0.114)	<0.001
A/A	27	0.252	67	0.626	4.963 (2.761–8.919)	<0.001
χ^2^ = 5.407; *p =* 0.020						
C	103	0.481	75	0.350	0.678 (0.487–0.944)	0.021
A	111	0.519	139	0.650	1.475 (1.060–2.054)	0.021
c.803+221C > A – *TPH1* (rs1800532)						
C/C	23	0.215	41	0.383	2.269 (1.240–4.150)	0.008
C/A	83	0.776	48	0.449	0.235 (0.130–0.426)	<0.001
A/A	1	0.009	18	0.168	21.438 (2.806–163.770)	0.003
χ^2^ = 0.014; *p =* 0.907						
C	129	0.602	130	0.607	1.028 (0.651–1.623)	0.907
A	85	0.397	84	0.393	0.973 (0.616–1.537)	0.907
c.-173A > T – *TPH1* (rs10488682)						
T/T	57	0.533	64	0.598	1.306 (0.759–2.245)	0.335
A/T	47	0.439	40	0374	0.762 (0.441–1.317)	0.330
A/A	3	0.028	3	0.028	1.000 (0.197–5.069)	1.000
χ^2^ = 0.753; *p =* 0.386						
T	161	0.752	168	0.785	1.240 (0.762–2.019)	0.387
A	53	0.248	46	0.215	0.806 (0.495–1.313)	0.387
c.-1668T > A – *TPH1* (rs623580)						
T/T	53	0.495	22	0.206	0.264 (0.144–0.482)	<0.001
T/A	45	0.421	62	0.579	1.898 (1.103–3.267)	0.021
A/A	9	0.084	23	0.215	2.981 (1.308–6.796)	0.009
χ^2^ = 21,356; *p <* 0.001						
T	151	0.706	106	0.495	0.373 (0.240–0.581)	<0.001
A	63	0.294	108	0.505	2.681 (1.722–4.173)	<0.001
c. – 844G > T – *TPH2* (rs4570625)						
G/G	8	0.075	78	0.729	33.284 (14.411–76.874)	<0.001
G/T	97	0.907	29	0.271	0.038 (0.018–0.084)	<0.001
T/T	2	0.019	0	0	-	-
χ^2^ = 107.455; *p <* 0.001						
G	113	0.528	185	0.864	32.746 (14.191–75.563)	<0.001
T	101	0.472	29	0.136	0.031 (0.013–0.071)	<0.001
c.-1449C > A – *TPH2* (rs7963803)						
C/C	65	0.607	98	0.916	7.036 (3.208–15.429)	<0.001
C/A	34	0.318	1	0.009	0.020 (0.003–0.151)	<0.001
A/A	8	0.075	8	0.075	1.000 (0.361–2.770)	1.000
χ^2^ = 14.794; *p <* 0.001						
C	164	0.766	197	0.921	2.596 (1.525–4.418)	<0.001
A	50	0.234	17	0.079	0.385 (0.226–0.656)	<0.001
c.*46G > A – *KAT1* (rs10988134)						
A/A	5	0.047	1	0.009	0.192 (0.022–1.676)	0.136
A/G	35	0.327	37	0.346	1.087 (0.617–1.918)	0.772
G/G	67	0.626	69	0.645	1.084 (0.621–1.892)	0.776
χ^2^ = 0.572; *p =* 0.450						
A	45	0.210	39	0.182	0.826 (0.503–1.356)	0.450
G	169	0.790	175	0.818	1.210 (0.737–1.987)	0.450
c.975-7T > C – *KAT2* (rs1480544)						
C/C	29	0.271	72	0.673	5.533 (3.076–9.954)	<0.001
T/C	59	0.551	4	0.037	0.032 (0.011–0.092)	<0.001
T/T	19	0.178	31	0.290	1.889 (0.988–3.613)	0.054
χ^2^ = 6.986; *p =* 0.008						
C	117	0.547	148	0.692	1.576 (1.119–2.219)	0.009
T	97	0.453	66	0.308	0.634 (0.451–0.893)	0.009
c.-1849C > A – *IDO1* (rs3824259)						
C/C	31	0.290	25	0.234	0.747 (0.405–1.379)	0.351
C/A	47	0.439	59	0.551	1.569 (0.915–2.691)	0.102
A/A	29	0.271	23	0.215	0.736 (0.393–1.380)	0.340
χ^2^ = 214.000; *p =* 0.429						
C	109	0.509	109	0.509	1.000 (0.686–1.458)	1.000
A	105	0.491	105	0.491	1.000 (0.686–1.458)	1.000
c. -1493G > C – *IDO1* (rs10089084)						
G/G	12	0.112	44	0.411	5.529 (2.709–11.284)	<0.001
G/C	55	0.514	54	0.504	0.963 (0.564–1.646)	0.891
C/C	40	0.374	9	0.084	0.154 (0.070–0.338)	<0.001
χ^2^ = 40.577; *p <* 0.001						
G	79	0.369	142	0.664	4.016 (2.487–6.484)	<0.001
C	135	0.631	72	0.336	0.249 (0.154–0.402)	<0.001

## Supplementary Materials

The following are available online at https://www.mdpi.com/2077-0383/8/12/2133/s1, Table S1: Haplotypes of TPH1, TPH2 and IDO1 and the risk of stroke. Table S2: Gene-gene interactions of studied polymorphisms and the risk of stroke.
