# Supplementary material for: Variation of Genes Encoding Tryptophan Catabolites Pathway Enzymes in Stroke
Source: J Clin Med. 2019 Dec 3;8(12):2133. doi: 10.3390/jcm8122133 (PMC6947300; doi:10.3390/jcm8122133)
Supplement: Supplementary file 1 [file jcm-08-02133-s001.pdf]

Table 1. Haplotypes of TPH1, TPH2 and IDO1 and the risk of stroke.

Haplotype	Control (n = 107)		Stroke (n = 107)		Crude OR (95% CI)	P
	Number	Frequency	Number	Frequency		
c.804-7C>A – *TPH1* (rs1799913) and c.803+221C>A – *TPH1* (rs1800532)						
CC	141	0.33	149	0.35	1.087 (0.819-1.443)	0.564
CA	117	0.27	111	0.26	0.931 (0.687-1.260)	0.643
AC	65	0.15	1	0.01	0.013 (0.002-0.095)	<0.0001
AA	105	0.25	167	0.39	1.968 (1.468-2.640)	<0.0001
c.804-7C>A – *TPH1* (rs1799913) and c.-173A>T –TPH1 (rs10488682)						
AA	42	0.10	35	0.08	0.819 (0.512-1.310)	0.404
CT	142	0.33	93	0.22	0.559 (0.412-0.759)	0.0002
CA	64	0.15	57	0.13	0.874 (0.595-1.284)	0.492
AT	180	0.42	243	0.57	1.810 (1.380-2.373)	<0.0001
c.804-7C>A – *TPH1* (rs1799913) and c.-1668T>A - TPH1 (rs623580)						
TT	135	0.32	91	0.21	0.586 (0.431-0.800)	0.001
CA	71	0.17	169	0.39	3.281 (2.382-4.519)	<0.0001
AT	167	0.39	121	0.28	0.616 (0.463-0.820)	0.001
AA	55	0.13	47	0.11	0.837 (0.553-1.267)	0.399
c.-173A>T – *TPH1* (rs10488682) and c.803+221C>A – *TPH1* (rs1800532)						
AC	71	0.17	76	0.18	1.086 (0.761-1.549)	0.651
AA	35	0.08	16	0.04	0.436 (0.238-0.801)	0.007
TC	187	0.44	184	0.43	0.972 (0.742-1.274)	0.836
TA	135	0.08	152	0.36	1.195 (0.900-1.588)	0.219
c.-1668T>A – *TPH1* (rs623580) and c.803+221C>A – *TPH1* (rs1800532)						
TC	176	0.41	91	0.21	0.387 (0.286-0.523)	<0.0001
TA	126	0.29	121	0.28	0.945(0.703-1.270)	0.7061
AC	82	0.19	169	0.39	2.753 (2.021-3.750)	<0.0001
AA	44	0.10	47	0.11	1.077 (0.697-1.663)	0.7394
c.-173A>T – *TPH1* (rs10488682) and c.-1668T>A – *TPH1* (rs623580)						
AT	89	0.21	29	0.07	0.277 (0.178-0.431)	<0.0001
AA	17	0.04	63	0.15	4.173 (2.398-7.261)	<0.0001
TT	213	0.50	183	0.43	0.754 (0.576-0.987)	0.0399
TA	109	0.25	153	0.36	1.628 (1.214-2.185)	0.0011
c.-1449C>A – *TPH2* (rs7963803) and c.-844G>T – *TPH2* (rs4570625)						
CG	173	0.40	353	0.82	6.938 (5.062-9.509)	<0.0001
CT	155	0.36	41	010	0.187 (0.128-0.272)	<0.0001
AG	53	0.12	17	004	0.293 (0.167-0.514)	<0.0001
AT	47	0.11	17	0.04	0.335 (0.189-0.594)	0.0002
c.-1849C>A – *IDO1* (rs3824259) and c. -1493G>C – *IDO1* (rs10089084)						
CG	62	0.14	175	0.41	4.083 (2.933-5.686)	<0.0001
CC	156	0.36	43	0.10	0.195 (0.134-0.282)	<0.0001
AG	96	0.22	109	0.25	1.182(0.863-1.618)	0.298
AC	114	0.27	101	0.24	0.851 (0.624-1.159)	0.306

Table 2. Gene-gene interactions of studied polymorphisms and the risk of stroke.

Combined genotype	Control (n = 107)		Stroke (n = 107)		Crude OR (95% CI)	p
	Number	Frequency	Number	Frequency		
**c.804-7C>A – *TPH1* (rs1799913) and c.-844G>T – *TPH2* (rs4570625)**						
**C/C-G/G**	**3**	**0.028**	**21**	**0.196**	**8.465 (2.442 – 29.340)**	**<0.001**
C/C-G/T	20	0.187	14	0.131	0.655 (0.312 – 1.377)	0.264
C/C-T/T	0	0	0	0	-	-
C/A-G/G	3	0.028	4	0.037	1.346 (0.294 – 6.165)	0.702
**C/A-G/T**	**52**	**0.486**	**1**	**0.009**	**0.010 (0.001 – 0.074)**	**<0.001**
C/A-T/T	2	0.019	0	0	-	-
**A/A-G/G**	**2**	**0.019**	**53**	**0.495**	**51.528 (12.093 – 219.550)**	**<0.001**
A/A-G/T	25	0.234	14	0.131	0.494 (0.241 – 1.013)	0.054
A/A-T/T	0	0	0	0	-	-
c.804-7C>A – *TPH1* (rs1799913) and c.-1449C>A – *TPH2* (rs7963803)						
**C/C-C/C**	**13**	**0.121**	**31**	**0.290**	**2.949 (1.443 – 6.027)**	**0.003**
C/C-C/A	7	0.065	0	0	-	-
C/C-A/A	3	0.028	4	0.037	1.346 (0.294 – 6.165)	0.702
**C/A-C/C**	**36**	**0.336**	**4**	**0.037**	**0.077 (0.026 – 0.225)**	**<0.001**

**C/A-C/A**	**18**	**0.168**	**0**	**0**	**-**	**-**
C/A-A/A	3	0.028	1	0.009	0.327 (0.034 – 3.195)	0.337
**A/A-C/C**	**16**	**0.150**	**63**	**0.589**	**8.143 (4.225 – 15.696)**	**<0.001**
**A/A-C/A**	**9**	**0.084**	**1**	**0.009**	**0.103 (0.013 – 0.826)**	**0.032**
A/A-A/A	2	0.019	3	0.028	-	-
c.803+221C>A – *TPH1* (rs1800532) and c.-844G>T – *TPH2* (rs4570625)						
**C/C-G/G**	**3**	**0.028**	**26**	**0.243**	**11.128 (3.253 – 38.065)**	**<0.001**
C/C-G/T	20	0.187	15	0.140	0.709 (0.342 – 1.473)	0.357
C/C-T/T	0	0	0	0	-	-
**C/A-G/G**	**5**	**0.047**	**36**	**0.336**	**10.344 (3.870 – 27.650)**	**<0.001**
**C/A-G/T**	**76**	**0.710**	**12**	**0.112**	**0.052 (0.025 – 0.107)**	**<0.001**
C/A-T/T	2	0.019	0	0	-	-
A/A-G/G	0	0	16	0.150	-	-
A/A-G/T	1	0.009	2	0.019	2.019 (0.180 – 22.606)	0.569
A/A-T/T	0	0	0	0	-	-
c.803+221C>A – *TPH1* (rs1800532) and c.-1449C>A – *TPH2* (rs7963803)						
**C/C-C/C**	**16**	**0.150**	**36**	**0.336**	**2.884 (1.482 – 5.611)**	**0.002**
C/C-C/A	6	0.056	0	0	-	-
C/C-A/A	1	0.009	5	0.047	5.196 (0.597 – 45.244)	0.136
C/A-C/C	48	0.449	46	0.430	0.972 (0.540 – 1.591)	0.783
**C/A-C/A**	**28**	**0.262**	**1**	**0.009**	**0.027 (0.004 – 0.200)**	**<0.001**
C/A-A/A	7	0.065	1	0.009	0.135 (0.016 – 1.115)	0.063
**A/A-C/C**	**1**	**0.009**	**16**	**0.10**	**18.637 (2.424 – 143.277)**	**0.005**
A/A-C/A	0	0	0	0	-	-
A/A-A/A	0	0	2	0.019	-	-
c.-173A>T – *TPH1* (rs10488682) and c.-844G>T – *TPH2* (rs4570625)						
**T/T-G/G**	**3**	**0.028**	**50**	**0.467**	**30.409 (9.077 – 101.874)**	**<0.001**
**T/T-G/T**	**53**	**0.495**	**14**	**0.131**	**0.153 (0.078 – 0.302)**	**<0.001**
T/T-T/T	1	0.009	0	0	-	-
**A/T-G/G**	**5**	**0.047**	**27**	**0.252**	**6.885 (2.538 – 18.680)**	**<0.001**
**A/T-G/T**	**41**	**0.383**	**13**	**0.121**	**0.223 (0.111 – 0.448)**	**<0.001**
A/T-T/T	1	0.009	0	0	-	-
A/A-G/G	0	0	1	0.009	-	-
A/A-G/T	3	0.028	2	0.019	0.660 (0.108 – 4.033)	0.653
A/A-T/T	0	0	0	0	-	-
c.-173A>T – *TPH1* (rs10488682) and c.-1449C>A – *TPH2* (rs7963803)						
**T/T-C/C**	**30**	**0.280**	**58**	**0.542**	**3.038 (1.722 – 5.361)**	**<0.001**
T/T-C/A	19	0.178	0	0	-	-
T/T-A/A	8	0.075	6	0.056	0.735 (0.246 – 2.196)	0.582
A/T-C/C	34	0.318	39	0.364	1.231 (0.699 – 2.169)	0.471
**A/T-C/A**	**13**	**0.121**	**1**	**0.009**	**0.068 (0.009 – 0.531)**	**0.010**
A/T-A/A	0	0	0	0	-	-
A/A-C/C	1	0.009	1	0.009	1.000 (0.067 – 16.197)	1.000
A/A-C/A	2	0.019	0	0	-	-
A/A-A/A	0	0	2	0.019	-	-
c.-1668T>A – *TPH1* (rs623580) and c.-844G>T – *TPH2* (rs4570625)						
**T/T-G/G**	**2**	**0.019**	**18**	**0.168**	**10.618 (2.398 – 47.015)**	**0.002**
**T/T-G/T**	**50**	**0.467**	**4**	**0.037**	**0.044 (0.015 – 0.129)**	**<0.001**
T/T-T/T	1	0.009	0	0	-	-
**T/A-G/G**	**6**	**0.056**	**43**	**0.402**	**11.310 (4.554 – 28.090)**	**<0.001**
**T/A-G/T**	**38**	**0.355**	**19**	**0.178**	**0.392 (0.208 – 0.739)**	**0.004**
T/A-T/T	1	0.009	0	0	-	-
**A/A-G/G**	**0**	**0**	**17**	**0.159**	**-**	**-**
A/A-G/T	9	0.084	6	0.056	0.647 (0.222 – 1.885)	0.425
A/A-T/T	0	0	0	0	-	-
c.-1668T>A – *TPH1* (rs623580) and c.-1449C>A – *TPH2* (rs7963803)						
**T/T-C/C**	**34**	**0.318**	**18**	**0.168**	**0.434 (0.227 – 0.832)**	**0.012**
T/T-C/A	18	0.168	0	0	-	-
T/T-A/A	1	0.009	4	0.037	4.117 (0.452 – 37.451)	0.209
**T/A-C/C**	**25**	**0.234**	**57**	**0.533**	**3.739 (2.079 – 6.726)**	**<0.001**
**T/A-C/A**	**16**	**0.150**	**1**	**0.009**	**0.053 (0.007 – 0.412)**	**0.005**
T/A-A/A	4	0.037	4	0.037	1.000 (0.244 – 4.106)	1.000
**A/A-C/C**	**6**	**0.056**	**23**	**0.215**	**4.609 (1.793 – 11.846)**	**0.002**
A/A-C/A	0	0	0	0	-	-
A/A-A/A	3	0.028	0	0	-	-
c.*456G>A (rs10988134) – *KAT1* and c. -1849C>A – *IDO1* (rs3824259)						

A/A-C/C	1	0.009	0	0	-	-
A/A-C/A	1	0.009	1	0.009	1.000 (0.062-16.197)	1.000
A/A-A/A	3	0.028	0	0	-	-
A/G-C/C	6	0.056	9	0.084	1.546 (0.530-4.506)	0.425
A/G-C/A	19	0.178	17	0.159	0.875 (0.427-1.792)	0.715
A/G-A/A	10	0.093	11	0.013	1.111 (0.451-2.738)	0.818
G/G-C/C	24	0.224	16	0.150	0.608 (0.302-1.223)	0.163
**G/G-C/A**	**27**	**0.252**	**41**	**0.383**	**1.841 (1.025-3.304)**	**0.041**
G/G-A/A	16	0.150	12	0.112	0.718 (0.322-1.602)	0.419
c.975-7T>C– *KAT2* (rs1480544) and– c. -1849C>A *IDO1* (rs3824259)						
C/C-CC	9	0.084	16	0.150	1.915 (0.806-4.547)	0.141
**C/C-CA**	**14**	**0.131**	**42**	**0.393**	**4.292 (2.168-8.497)**	**<0.001**
C/C-AA	6	0.056	14	0.131	2.534 (0.935-6.868)	0.068
T/C-CC	17	0.159	0	0	-	-
**T/C-CA**	**26**	**0.243**	**1**	**0.009**	**0.029 (0.004-0.221)**	**<0.001**
**T/C-AA**	**16**	**0.150**	**3**	**0.028**	**0.164 (0.046-0.581)**	**0.005**
T/T-CC	5	0.047	9	0.084	1.873 (0.607-5.787)	0.275
T/T-CA	7	0.065	16	0.150	2.512 (0.989-6.382)	0.053
T/T-AA	7	0.065	6	0.561	0.849 (0.276-2.614)	0.775
c. -1493G>C – *IDO1* (rs10089084) and c.*456G>A– *KAT1* (rs10988134)						
**G/G-G/G**	**8**	**0.075**	**29**	**0.271**	**4.601 (1.992-10.626)**	**<0.001**
**G/G-G/A**	**4**	**0.037**	**15**	**0.140**	**4.198 (1.345-13.104)**	**0.013**
G/G-A/A	0	0	0	0	-	-
G/C-G/G	35	0.327	37	0.346	1.087 (0.617-1.918)	0.772
G/C-G/A	18	0.168	16	0.150	0.869 (0.417-1.811)	0.709
G/C-A/A	2	0.190	1	0.009	0.495 (0.044-5.545)	0.569
**C/C-G/G**	**24**	**0.224**	**3**	**0.028**	**0.100 (0.029-0.343)**	**<0.001**
C/C-G/A	13	0.121	6	0.056	0.430 (0.157-1.176)	0.100
C/C-A/A	3	0.028	0	0	-	-
c.*456G>A– *KAT1* (rs10988134) and c.975-7T>C– *KAT2* (rs1480544)						
G/G-T/T	10	0.093	20	0.187	2.230 (0.990-5.025)	0.053
**G/G-T/C**	**41**	**0.383**	**2**	**0.019**	**0.031 (0.007-0.131)**	**<0.001**
**G/G-C/C**	**16**	**0.150**	**47**	**0.439**	**4.455 (2.316-8.571)**	**<0.001**
G/A-T/T	9	0.0841	11	0.103	1.248 (0.945-3.146)	0.639
**G/A-T/C**	**14**	**0.131**	**2**	**0.019**	**0.127 (0.028-0.571)**	**0.007**
**G/A-C/C**	**12**	**0.112**	**24**	**0.224**	**2.289 (1.078-4.860)**	**0.031**
A/A-T/T	0	0	0	0	-	-
A/A-T/C	4	0.037	0	0	-	-
A/A-C/C	1	0.009	1	0.009	1.000 (0.062-16.197)	1.000
c. -1493G>C – *IDO1* (rs10089084) and c.975-7T>C– *KAT2* (rs1480544)						
**G/G-T/T**	**4**	**0.0.037**	**16**	**0.150**	**4.527 (1.461-14.035)**	**0.009**
G/G-T/C	6	0.056	1	0.009	0.159 (0.019-1.342)	0.091
**G/G-C/C**	**2**	**0.019**	**27**	**0.252**	**17.719 (4.092-76.715)**	**<0.001**
G/C-T/T	9	0.084	14	0.131	1.639 (0.677-3.968)	0.273
**G/C-T/C**	**31**	**0.290**	**3**	**0.028**	**0.071 (0.021-0.240)**	**<0.001**
**G/C-C/C**	**15**	**0.140**	**37**	**0.346**	**3.242 (1.650-6.372)**	**<0.001**
C/C-T/T	6	0.056	1	0.009	0.159 (0.019-1.342)	0.091
C/C-T/C	22	0.206	0	0	-	-
C/C-C/C	12	0.112	8	0.075	0.640 (0.250-1.634)	0.351
c. -1849C>A – *IDO1* (rs3824259) and c.804-7C>A - *TPH1* (rs1799913)						
C/C-C/C	8	0.075	10	0.093	1.276 (0.483-3.369)	0.623
**C/C-C/A**	**17**	**0.159**	**1**	**0.009**	**0.050 (0.007-0.383)**	**0.004**
C/C-A/A	6	0.056	14	0.131	2.534 (0.935-6.868)	0.068
C/A-C/C	9	0.084	18	0.168	2.202 (0.941-5.153)	0.069
**C/A-C/A**	**30**	**0.280**	**4**	**0.037**	**0.100 (0.034-0.295)**	**<0.001**
**C/A-A/A**	**8**	**0.075**	**37**	**0.346**	**6.541 (2.872-14.899)**	**<0.001**
A/A-C/C	6	0.056	7	0.065	1.178 (0.383-3.629)	0.775
A/A-C/A	10	0.093	0	0	-	-
A/A-A/A	13	0.121	16	0.150	1.271 (0.579-2.792)	0.550
c. -1849C>A – *IDO1* (rs3824259) and c.-844G>T – *TPH2* (rs4570625)						
**C/C-G/G**	**5**	**0.047**	**20**	**0.187**	**4.690 (1.690-13.017)**	**0.003**
**C/C-G/T**	**26**	**0.243**	**5**	**0.047**	**0.153 (0.056-0.415)**	**<0.001**
C/C-T/T	0	0	0	0	-	-

**C/A-G/G**	**1**	**0.009**	**41**	**0.383**	**65.848 (8.846-490.165)**	**<0.001**
**C/A-G/T**	**44**	**0.411**	**18**	**0.168**	**0.290 (0.153-0.547)**	**<0.001**
C/A-T/T	2	0.019	0	0	-	-
**A/A-G/G**	**2**	**0.019**	**17**	**0.159**	**9.917 (2.231-44.088)**	**0.003**
**A/A-G/T**	**27**	**0.252**	**6**	**0.056**	**0.176 (0.069-0.447)**	**<0.001**
A/A-T/T	0	0	0	0	-	-
c. -1849C>A – *IDO1* (rs3824259) and c.803+221C>A – *TPH1* (rs1800532)						
C/C-C/C	7	0.065	12	0.112	1.805 (0.682-4.777)	0.235
**C/C-C/A**	**24**	**0.224**	**11**	**0.103**	**0.396 (0.183-0.857)**	**0.019**
C/C-A/A	0	0	2	0.019	-	-
**C/A-C/C**	**10**	**0.093**	**22**	**0.206**	**2.511 (1.126-5.600)**	**0.025**
C/A-C/A	37	0.346	29	0.271	0.703 (0.392-1.261)	0.237
C/A-A/A	0	0	8	0.075	-	-
A/A-C/C	6	0.056	7	0.065	1.178 (0.383-3.629)	0.775
**A/A-C/A**	**22**	**0.206**	**8**	**0.075**	**0.312 (0.132-0.737)**	**0.008**
**A/A-A/A**	**1**	**0.009**	**8**	**0.075**	**8.566 (1.052-69.727)**	**0.045**
c. -1849C>A – *IDO1* (rs3824259) and c.-1449C>A – *TPH2* (rs7963803)						
C/C-C/C	19	0.178	23	0.215	1.268 (0.644-2.496)	0.492
**C/C-C/A**	**9**	**0.084**	**1**	**0.009**	**0.103 (0.013-0.826)**	**0.032**
C/C-A/A	3	0.028	1	0.009	0.327 (0.034-3.195)	0.337
**C/A-C/C**	**32**	**0.299**	**55**	**0.514**	**2.479 (1.414-4.345)**	**0.002**
C/A-C/A	12	0.112	0	0	-	-
C/A-A/A	3	0.028	4	0.037	1.346 (0.294-6.165)	0.702
A/A-C/C	14	0.131	20	0.187	1.527 (0.726-3.210)	0.264
A/A-C/A	13	0.121	0	0	-	-
A/A-A/A	2	0.019	3	0.028	1.514 (0.248-9.250)	0.653
c. -1849C>A – *IDO1* (rs3824259) and c.-173A>T – *TPH1* (rs10488682)						
C/C-A/A	1	0.009	1	0.009	1.000 (0.062-16.197)	1.000
C/C-A/T	12	0.112	11	0.103	0.907 (0.382-2.156)	0.825
C/C-T/T	18	0.168	13	0.121	0.684 (0.317-1.477)	0.333
C/A-A/A	1	0.009	0	0	-	-
C/A-A/T	24	0.224	24	0.224	1.000 (0.526-1.901)	1.000
**C/A-T/T**	**22**	**0.206**	**35**	**0.327**	**1.878 (1.012-3.487)**	**0.046**
A/A-A/A	1	0.009	2	0.190	2.019 (0.180-22.606)	0.569
A/A-A/T	11	0.103	5	0.047	0.428 (0.143-1.277)	0.128
A/A-T/T	17	0.159	16	0.150	0.931 (0.443-1.955)	0.840
c. -1849C>A – *IDO1* (rs3824259) and c.-1668T>A – *TPH1* (rs623580)						
**C/C-T/T**	**15**	**0.140**	**2**	**0.019**	**0.117 (0.026-0.524)**	**0.005**
C/C-T/A	12	0.112	18	0.168	1.601 (0.730-3.512)	0.240
C/C-A/A	4	0.037	5	0.047	1.262 (0.330-4.835)	0.734
**C/A-T/T**	**22**	**0.206**	**10**	**0.093**	**0.398 (0.179-0.888)**	**0.028**
**C/A-T/A**	**22**	**0.206**	**36**	**0.336**	**1.959 (1.057-3.631)**	**0.033**
**C/A-A/A**	**3**	**0.028**	**13**	**0.121**	**4.794 (1.325-17.347)**	**0.017**
A/A-T/T	16	0.150	10	0.093	0.586 (0.253-1.359)	0.213
A/A-T/A	11	0.103	8	0.075	0.705 (0.272-1.829)	0.473
A/A-A/A	2	0.019	5	0.047	2.574 (0.488-13.566)	0.265
c. -1493G>C – *IDO1* (rs10089084) and c.804-7C>A – *TPH1* (rs1799913)						
**G/G-C/C**	**1**	**0.009**	**18**	**0.168**	**21.438 (2.806-163.770)**	**0.003**
G/G-C/A	5	0.047	0	0	-	-
**G/G-A/A**	**6**	**0.056**	**26**	**0.243**	**5.403 (2.122-13.758)**	**<0.001**
G/C-C/C	12	0.112	14	0.131	1.192 (0.524-2.712)	0.676
**G/C-C/A**	**31**	**0.290**	**5**	**0.047**	**0.120 (0.045-0.323)**	**<0.001**
**G/C-A/A**	**12**	**0.112**	**35**	**0.327**	**3.848 (1.866-7.935)**	**<0.001**
C/C-C/C	10	0.093	3	0.028	0.280 (0.075-1.047)	0.059
C/C-C/A	21	0.196	0	0	-	-
C/C-A/A	9	0.084	6	0.056	0.647 (0.222-1.885)	0.425
c. -1493G>C – *IDO1* (rs10089084) and c.-844G>T – *TPH2* (rs4570625)						
G/G-G/G	0	0	32	0.299	-	-
G/G-G/T	12	0.112	12	0.112	1.000 (0.428-2.338)	1.000
G/G-T/T	0	0	0	0	-	-
**G/C-G/G**	**5**	**0.047**	**40**	**0.374**	**12.179 (4.573-32.433)**	**<0.001**
**G/C-G/T**	**49**	**0.458**	**14**	**0.131**	**0.178 (0.091-0.351)**	**<0.001**
G/C-T/T	1	0.009	0	0	-	-

C/C-G/G	3	0.028	6	0.056	2.059 (0.501-8.458)	0.316
**C/C-G/T**	**36**	**0.336**	**3**	**0.028**	**0.057 (0.017-0.192)**	**<0.001**
C/C-T/T	1	0.009	0	0	-	-
c. -1493G>C – *IDO1* (rs10089084) and c.803+221C>A – *TPH1* (rs1800532)						
**G/G-C/C**	**1**	**0.009**	**19**	**0.178**	**22.886 (3.004-174.371)**	**0.003**
**G/G-C/A**	**11**	**0.103**	**22**	**0.206**	**2.259 (1.035-4.930)**	**0.041**
G/G-A/A	0	0	3	0.028	-	-
G/C-C/C	11	0.103	19	0.178	1.884 (0.849-4.181)	0.119
**G/C-C/A**	**44**	**0.411**	**24**	**0.224**	**0.414 (0.228-0.751)**	**0.004**
G/C-A/A	0	0	11	0.103	-	-
**C/C-C/C**	**11**	**0.103**	**3**	**0.028**	**0.252 (0.068-0.930)**	**0.039**
**C/C-C/A**	**28**	**0.262**	**2**	**0.019**	**0.054 (0.012-0.232)**	**<0.001**
C/C-A/A	1	0.009	4	0.037	4.117 (0.452-37.451)	0.209
c. -1493G>C – *IDO1* (rs10089084) and c.-1449C>A – *TPH2* (rs7963803)						
**G/G-C/C**	**7**	**0.065**	**41**	**0.383**	**8.874 (3.756-20.966)**	**<0.001**
G/G-C/A	3	0.028	1	0.009	0.327 (0.034-3.195)	0.337
G/G-A/A	2	0.019	2	0.019	1.000 (0.138-7.232)	1.000
**G/C-C/C**	**28**	**0.262**	**48**	**0.449**	**2.295 (1.291-4.080)**	**0.005**
G/C-C/A	23	0.215	0	0	-	-
G/C-A/A	4	0.037	6	0.056	1.530 (0.419-5.583)	0.520
**C/C-C/C**	**30**	**0.280**	**9**	**0.084**	**0.236 (0.106-0.526)**	**<0.001**
C/C-C/A	8	0.075	0	0	-	-
C/C-A/A	2	0.019	0	0	-	-
c. -1493G>C – *IDO1* (rs10089084) and c.-173A>T – *TPH1* (rs10488682)						
G/G-A/A	0	0	3	0.028	-	-
**G/G-A/T**	**4**	**0.037**	**20**	**0.187**	**5.920 (1.949-17.976)**	**0.002**
**G/G-T/T**	**8**	**0.075**	**21**	**0.196**	**3.022 (1.274-7.170)**	**0.012**
G/C-A/A	2	0.019	0	0	-	-
G/C-A/T	26	0.243	17	0.159	0.588 (0.298-1.163)	0.127
G/C-T/T	27	0.252	37	0.346	1.566 (0.867-2.827)	0.137
C/C-A/A	1	0.009	0	0	-	-
**C/C-A/T**	**17**	**0.159**	**3**	**0.028**	**0.153 (0.043-0.538)**	**0.003**
**C/C-T/T**	**22**	**0.206**	**6**	**0.056**	**0.230 (0.089-0.592)**	**0.002**
c. -1493G>C – *IDO1* (rs10089084) and c.-1668T>A – *TPH1* (rs623580)						
G/G-T/T	7	0.065	5	0.047	0.700 (0.215-2.280)	0.554
**G/G-T/A**	**5**	**0.047**	**30**	**0.280**	**7.948 (2.948-21.431)**	**<0.001**
G/G-A/A	0	0	9	0.084	-	-
**G/C-T/T**	**28**	**0.262**	**13**	**0.121**	**0.390 (0.189-0.804)**	**0.011**
G/C-T/A	22	0.206	30	0.280	1.505 (0.801-2.828)	0.204
G/C-A/A	5	0.047	11	0.103	2.337 (0.783-6.975)	0.128
**C/C-T/T**	**18**	**0.268**	**4**	**0.037**	**0.192 (0.063-0.588)**	**0.004**
**C/C-T/A**	**18**	**0.268**	**2**	**0.019**	**0.094 (0.021-0.417)**	**0.002**
C/C-A/A	4	0.037	3	0.028	0.743 (0.162-3.401)	0.702
c.*456G>A (rs10988134) – *KAT1* and c.804-7C>A – *TPH1* (rs1799913)						
G/G-C/C	17	0.159	19	0.178	1.143 (0.558-2.342)	0.715
**G/G-C/A**	**34**	**0.318**	**4**	**0.037**	**0.083 (0.028-0.245)**	**<0.001**
**G/G-A/A**	**16**	**0.150**	**46**	**0.430**	**4.289 (2.228-8.255)**	**<0.001**
**G/A-C/C**	**5**	**0.047**	**16**	**0.150**	**3.587 (1.264-10.180)**	**0.016**
**G/A-C/A**	**21**	**0.196**	**1**	**0.009**	**0.039 (0.005-0.293)**	**0.002**
**G/A-A/A**	**9**	**0.084**	**20**	**0.1987**	**2.503 (1.083-5.787)**	**0.032**
A/A-C/C	1	0.009	0	0	-	-
A/A-C/A	2	0.019	0	0	-	-
A/A-A/A	2	0.019	1	0.009	0.495 (0.044-5.545)	0.569
c.*456G>A (rs10988134) – *KAT1* and c.-844G>T – *TPH2* (rs4570625)						
**G/G-G/G**	**8**	**0.075**	**51**	**0.477**	**11.270 (4.993-25.440)**	**<0.001**
**G/G-G/T**	**58**	**0.542**	**18**	**0.168**	**0.171 (0.091-0.322)**	**<0.001**
G/G-T/T	1	0.009	0	0	-	-
G/A-G/G	0	0	26	0.243	-	-
**G/A-G/T**	**34**	**0.318**	**11**	**0.103**	**0.246 (0.117-0.518)**	**<0.001**
G/A-T/T	1	0.009	0	0	-	-
A/A-G/G	0	0	1	0.009	-	-
A/A-G/T	5	0.047	0	0	-	-
A/A-T/T	0	0	0	0	-	-

c.*456G>A (rs10988134) – *KAT1* and c.803+221C>A – *TPH1* (rs1800532)						
G/G-C/C	16	0.150	22	0.206	1.472 (0.725-2.990)	0.285
**G/G-C/A**	**50**	**0.467**	**34**	**0.318**	**0.531 (0.304-0.926)**	**0.026**
**G/G-A/A**	**1**	**0.009**	**13**	**0.121**	**14.660 (1.882-114.195)**	**0.010**
**G/A-C/C**	**6**	**0.056**	**18**	**0.168**	**3.404 (1.295-8.953)**	**0.013**
**G/A-C/A**	**29**	**0.271**	**14**	**0.131**	**0.405 (0.200-0.820)**	**0.012**
G/A-A/A	0	0	5	0.047	-	-
A/A-C/C	1	0.009	1	0.009	1.000 (0.062-16.197)	1.000
A/A-C/A	4	0.037	0	0	-	-
A/A-A/A	0	0	0	0	-	-
c.*456G>A (rs10988134) – *KAT1* and c.-1449C>A – *TPH2* (rs7963803)						
**G/G-C/C**	**38**	**0.355**	**60**	**0.561**	**2.318 (1.337-4.018)**	**0.003**
**G/G-C/A**	**26**	**0.243**	**1**	**0.009**	**0.029 (0.004-0.221)**	**<0.001**
G/G-A/A	3	0.028	8	0.075	2.801 (0.722-10.862)	0.136
**G/A-C/C**	**23**	**0.215**	**37**	**0.346**	**1.930 (1.049-3.551)**	**0.034**
G/A-C/A	8	0.075	0	0	-	-
G/A-A/A	4	0.037	0	0	-	-
A/A-C/C	4	0.037	1	0.009	0.243 (0.027-2.210)	0.209
A/A-C/A	0	0	0	0	-	-
A/A-A/A	1	0.009	0	0	-	-
c.*456G>A (rs10988134) – *KAT1* and c.-173A>T – *TPH1* (rs10488682)						
G/G-A/A	32	0.299	42	0.393	1.514 (0.859-2.671)	0.152
G/G-A/T	33	0.308	25	0.234	0.684 (0.372-1.255)	0.220
G/G-T/T	2	0.019	2	0.019	1.000 (0.138-7.232)	1.000
G/A-A/A	22	0.206	21	0.196	0.943 (0.483-1.842)	0.865
G/A-A/T	12	0.112	15	0.140	1.291 (0.573-2.906)	0.538
G/A-T/T	1	0.009	1	0.009	1.000 (0.062-16.197)	1.000
A/A-A/A	3	0.028	1	0.009	0.327 (0.034-3.195)	0.337
A/A-A/T	2	0.019	0	0	-	-
A/A-T/T	0	0	0	0	-	-
c.*456G>A (rs10988134) – *KAT1* and c.-1668T>A – *TPH1* (rs623580)						
**G/G-T/T**	**37**	**0.346**	**16**	**0.150**	**0.333 (0.171-0.646)**	**0.001**
**G/G-T/A**	**27**	**0.252**	**44**	**0.411**	**2.069 (1.156-3.703)**	**0.014**
G/G-A/A	3	0.028	9	0.084	3.184 (0.873-12.104)	0.089
**G/A-T/T**	**15**	**0.140**	**5**	**0.047**	**0.301 (0.105-0.860)**	**0.025**
G/A-T/A	14	0.131	18	0.168	1.343 (0.631-2.863)	0.444
G/A-A/A	6	0.056	14	0.130	2.534 (0.935-6.868)	0.068
A/A-T/T	1	0.009	1	0.009	1.000 (0.062-16.197)	1.000
A/A-T/A	4	0.037	0	0	-	-
A/A-A/A	0	0	0	0	-	-
c.975-7T>C– *KAT2* (rs1480544) and c.804-7C>A – *TPH1* (rs1799913)						
T/T-C/C	3	0.028	9	0.084	3.184 (0.837-12.104)	0.089
T/T-C/A	12	0.112	0	0	-	-
**T/T-A/A**	**4**	**0.037**	**22**	**0.206**	**6.665 (2.211-20.090)**	**<0.001**
T/C-C/C	16	0.150	0	0	-	-
T/C-C/A	32	0.299	0	0	-	-
T/C-A/A	11	0.103	4	0.037	0.339 (0.104-1.100)	0.072
**C/C-C/C**	**4**	**0.037**	**26**	**0.243**	**8.265 (2.773-24.637)**	**<0.001**
C/C-C/A	13	0.121	5	0.047	0.354 (0.122-1.032)	0.057
**C/C-A/A**	**12**	**0.112**	**41**	**0.383**	**4.918 (2.404-10.062)**	**<0.001**
c.975-7T>C– *KAT2* (rs1480544) and c.-844G>T – *TPH2* (rs4570625)						
**T/T-G/G**	**1**	**0.009**	**24**	**0.224**	**30.651 (4.062-231.263)**	**<0.001**
**T/T-G/T**	**17**	**0.159**	**7**	**0.065**	**0.371 (0.147-0.935)**	**0.035**
T/T-T/T	1	0.009	0	0	-	-
T/C-G/G	4	0.037	4	0.037	1.000 (0.244-4.106)	1.000
T/C-G/T	55	0.514	0	0	-	-
T/C-T/T	0	0	0	0	-	-
**C/C-G/G**	**3**	**0.028**	**50**	**0.467**	**30.409 (9.077-101.874)**	**<0.001**
C/C-G/T	25	0.234	22	0.206	0.849 (0.444-1.623)	0.621
C/C-T/T	1	0.009	0	0	-	-
c.975-7T>C– *KAT2* (rs1480544) and c.803+221C>A – *TPH1* (rs1800532)						
T/T-C/C	3	0.028	10	0.093	3.574 (0.955-13.372)	0.059
T/T-C/A	16	0.150	13	0.121	0.787 (0.358-1.727)	0.550

T/T-A/A	0	0	8	0.075	-	-
T/C-C/C	16	0.150	0	0	-	-
**T/C-C/A**	**42**	**0.393**	**3**	**0.028**	**0.045 (0.013-0.150)**	**<0.001**
T/C-A/A	1	0.009	1	0.009	1.000 (0.062-16.197)	1.000
**C/C-C/C**	**4**	**0.037**	**31**	**0.290**	**10.503 (3.557-31.011)**	**<0.001**
C/C-C/A	25	0.234	32	0.299	1.399 (0.761-2.575)	0.280
C/C-A/A	0	0	9	0.084	-	-
c.975-7T>C– *KAT2* (rs1480544) and c.-1449C>A – *TPH2* (rs7963803)						
**T/T-C/C**	**10**	**0.093**	**27**	**0.252**	**3.274 (1.495-7.167)**	**0.003**
T/T-C/A	7	0.065	0	0	-	-
T/T-A/A	2	0.019	4	0.037	2.039 (0.365-11.375)	0.417
**T/C-C/C**	**41**	**0.383**	**4**	**0.037**	**0.063 (0.021-0.183)**	**<0.001**
T/C-C/A	15	0.140	0	0	-	-
T/C-A/A	3	0.028	0	0	-	-
**C/C-C/C**	**14**	**0.131**	**67**	**0.626**	**11.127 1.070)**	**<0.001**
**C/C-C/A**	**12**	**0.112**	**1**	**0.009**	**0.075 (0.010-0.585)**	**0.014**
C/C-A/A	3	0.028	4	0.037	1.346 (0.294-6.165)	0.702
c.975-7T>C– *KAT2* (rs1480544) and c.-173A>T – *TPH1* (rs10488682)						
T/T-A/A	1	0.009	2	0.019	2.019 (0.180-22.606)	0.569
T/T-A/T	5	0.047	2	0.019	0.389 (0.074-2.048)	0.265
**T/T-T/T**	**13**	**0.121**	**27**	**0.252**	**2.440 (1.181-5.042)**	**0.016**
T/C-A/A	2	0.019	0	0	-	-
T/C-A/T	32	0.299	0	0	-	-
**T/C-T/T**	**25**	**0.234**	**4**	**0.037**	**0.127 (0.043-0.381)**	**<0.001**
C/C-A/A	0	0	1	0.009	-	-
**C/C-A/T**	**10**	**0.093**	**38**	**0.355**	**5.342 (2.494-11.445)**	**<0.001**
**C/C-T/T**	**19**	**0.178**	**33**	**0.308**	**2.065 (1.085-3.931)**	**0.027**
c.975-7T>C– *KAT2* (rs1480544) and c.-1668T>A – *TPH1* (rs623580)						
T/T-T/T	10	0.093	11	0.103	1.111 (0.451-2.738)	0.818
**T/T-T/A**	**7**	**0.065**	**19**	**0.178**	**3.084 (1.238-7.683)**	**0.016**
T/T-A/A	2	0.019	1	0.009	0.495 (0.044-5.545)	0.569
**T/C-T/T**	**28**	**0.261**	**1**	**0.009**	**0.027 (0.004-0.200)**	**<0.001**
**T/C-T/A**	**25**	**0.234**	**3**	**0.028**	**0.095 (0.028-0.324)**	**<0.001**
T/C-A/A	6	0.056	0	0	-	-
C/C-T/T	15	0.140	10	0.093	0.623 (0.270-1.479)	0.290
**C/C-T/A**	**13**	**0.121**	**40**	**0.374**	**4.317 (2.144-8.691)**	**<0.001**
**C/C-A/A**	**1**	**0.009**	**22**	**0.206**	**27.435 (3.624-207.693)**	**0.001**
